# Triptan non-response in specialized headache care: cross-sectional data from the DMKG Headache Registry

**DOI:** 10.1186/s10194-023-01676-0

**Published:** 2023-10-10

**Authors:** Ruth Ruscheweyh, Gudrun Gossrau, Thomas Dresler, Tobias Freilinger, Stefanie Förderreuther, Charly Gaul, Torsten Kraya, Lars Neeb, Victoria Ruschil, Andreas Straube, Jörg Scheidt, Tim Patrick Jürgens

**Affiliations:** 1 Department of Neurology, LMU University Hospital, LMU Munich, Marchioninistr. 15, Munich, 81377 Germany; 2 German Migraine and Headache Society, Frankfurt, Germany; 3 Department of Psychosomatic Medicine and Psychotherapy, Technical University of Munich, Munich, Germany; 4 Interdisciplinary Pain Center, University Hospital and Faculty of Medicine Carl Gustav Carus, TU Dresden, Dresden, Germany; 5 LEAD Graduate School & Research Network, University of Tuebingen, Tuebingen, Germany; 6 Department of Psychiatry and Psychotherapy, Tuebingen Center for Mental Health, University Hospital of Tuebingen, Tuebingen, Germany; 7 German Center for Mental Health (DZPG), Partner Site Tuebingen, Tuebingen, Germany; 8 Department of Neurology, Klinikum Passau, Passau, Germany; 9 Headache Center Frankfurt, Frankfurt, Germany; 10 Department of Neurology, Hospital Sankt Georg Leipzig gGmbH, Leipzig, Germany; 11 Headache Center Halle, Department of Neurology, University Hospital Halle, Halle, Germany; 12 Helios Global Health, Friedrichstraße 136, Berlin, 10117 Germany; 13 Department of Neurology and Epileptology, Hertie Institute for Clinical Brain Research, Eberhard-Karls University Tübingen, Tübingen, Germany; 14 Institute for Information Systems, University of Applied Sciences Hof, Hof, Germany; 15 Department of Neurology, Headache Center North-East, University Medical Center Rostock, Rostock, Germany; 16 Department of Neurology, KMG Klinikum Güstrow, Güstrow, Germany

**Keywords:** Registry, Headache, Migraine, Germany, Acute headache treatment, Triptan failure, Patient-reported outcome measures

## Abstract

**Background:**

Triptans are effective for many migraine patients, but some do not experience adequate efficacy and tolerability. The European Headache Federation (EHF) has proposed that patients with lack of efficacy and/or tolerability of ≥ 2 triptans (‘triptan resistance’) could be considered eligible for treatment with the novel medications from the ditan and gepant groups. There is little data on the frequency of ‘triptan resistance’.

**Methods:**

We used patient self-report data from the German Migraine and Headache Society (DMKG) Headache Registry to assess triptan response and triptan efficacy and/or tolerability failure.

**Results:**

A total of 2284 adult migraine patients (females: 85.4%, age: 39.4 ± 12.8 years) were included. 42.5% (*n* = 970) had failed ≥ 1 triptan, 13.1% (*n* = 300) had failed ≥ 2 triptans (meeting the EHF definition of ‘triptan resistance’), and 3.9% (*n* = 88) had failed ≥ 3 triptans. Compared to triptan responders (current use, no failure, *n* = 597), triptan non-responders had significantly more severe migraine (higher frequency (*p* < 0.001), intensity (*p* < 0.05), and disability (*p* < 0.001)), that further increased with the level of triptan failure. Responders rates were highest for nasal and oral zolmitriptan, oral eletriptan and subcutaneous sumatriptan.

**Conclusion:**

In the present setting (specialized headache care in Germany), 13.1% of the patients had failed ≥ 2 triptans. Triptan failure was associated with increased migraine severity and disability, emphasizing the importance of establishing an effective and tolerable acute migraine medication. Acute treatment optimization might include switching to one of the triptans with the highest responder rates and/or to a different acute medication class.

**Trial registration:**

The DMKG Headache Registry is registered with the German Clinical Trials Register (DRKS 00021081).

**Supplementary Information:**

The online version contains supplementary material available at 10.1186/s10194-023-01676-0.

## Background

Triptans have been the most effective acute migraine medication for decades [1, 2]. However, not all patients benefit. Some have contraindications, especially vascular disorders or uncontrolled arterial hypertension [3, 4]. Of those being eligible for triptan treatment, 30 to 60% do not have a 2 h response (headache improvement) to a specific triptan, and up to 40% have recurrence after initial pain freedom [2]. While severe adverse events are extremely rare [5], fatigue, dizziness, paresthesias and chest tightness may result in discontinuation or limited use of triptans. Persistence with triptan treatment is low both internationally [6] and in Germany, where health insurance data show that 60% of migraine patients discontinue their triptan, often after the first prescription [3]. Efficacy and tolerability can be improved for part of the patients by switching to a different triptan [1]. Nonetheless, a significant unmet need remains in acute migraine treatment.

Recently, ditans (5HT-1F-receptor agonists) and gepants (small molecule calcitonin gene-related peptide (CGRP) receptor antagonists) have been developed for acute migraine therapy [7]. Both groups are effective also in triptan non-responders [8, 9]. Lasmiditan and rimegepant have been approved by the Food and Drug Administration (FDA) in the United States and the European Medicines Agency (EMA) and are becoming increasingly available. Considering the substantial price difference compared to triptans, a working definition of triptan failure is needed for an economic approach.

The European Headache Federation (EHF) has recently proposed consensus criteria for triptan response and failure [10]. Response to a (specific) triptan was defined as relief of headache and non-pain migraine symptoms between 2 and 24 h in 3 of 4 treated attacks and absence of meaningful adverse events. ‘Triptan resistant migraine’ was defined as failure (non-response) of at least 2 triptans and ‘triptan refractory migraine’ as failure of at least 3 triptans, including a subcutaneous formulation. The EHF consensus proposed that patients with failure of ≥ 2 triptans might be eligible for treatment with gepants or ditans. Similarly, the American Headache Society (AHS) proposed that use of gepants or ditans is appropriate in patients who have inadequate response to two or more oral triptans [11].

There are currently few data to estimate how frequent failure of one or several triptans is. A pooled analysis of rimegepant studies showed insufficient response to ≥ 2 triptans in 9.3% of the study population, but may be biased towards patients dissatisfied with their current acute migraine medication [8]. In addition, the rate of triptan failure is likely to be higher in severely affected populations.

Here, we used data from the German Migraine and Headache Society (DMKG) Headache Registry [12] to estimate the frequency of failure of one or several triptans in migraine patients from German headache centers and private practices. To better understand patterns of triptan response and failure, we also investigated how triptan responders and non-responders differed in headache severity and other parameters. In addition, we report the use of specific triptans as first-, second-, or third-line treatments, and proportions of response and efficacy vs. tolerability failure for specific triptans.

## Methods

The DMKG Headache Registry is conducted in accordance with the Declaration of Helsinki and was approved by the leading ethics committee of the Ludwig-Maximilians-University Munich (Nr. 20–004), and by the ethics committee of each participating center. The registry complies with European and German Data Protection laws and is registered with the German Clinical Trials Register (DRKS 00021081).

The DMKG Headache Registry has been recruiting since June 2020. Detailed methods have been published [12]. At the time of the present analysis (data closure May 12, 2023), 22 DMKG-accredited centers had contributed data (14 private practices, 8 outpatient clinic-based, see Appendix for a list). We included all adult patients having ≥ 1 completed physician visit and an ICHD-3 diagnosis [13] of migraine without or with aura or chronic migraine (*n* = 2284). For every patient, the last available visit within the registry was analyzed to maximize information on different triptans tried by the patient.

Before their first visit at the center and before each follow-up visit, patients provided detailed information about their headache, acute and preventive medication and concomitant disorders via a web application [12]. For past acute headache medication, patients indicated the reason for discontinuation (side effects, no effect or insufficient effect, found better medication, discontinuation advised by physician, not needed anymore because of headache improvement, other). For current acute headache medication, patients rated efficacy and tolerability on a 6-point Likert scale (efficacy: very good, good, moderate, some, little, none; tolerability: very good, good, somewhat good, somewhat poor, poor, very poor). During each visit, the treating physician provided the ICHD-3 diagnosis [13] and confirmed or corrected some of the core entries (such as headache and medication days per month, current acute and preventive medication). If a current acute medication was discontinued, it was transferred to past medication.

### Analysis

Statistical analysis was performed with R (version 4.3.0). Descriptive statistics include mean ± standard deviation, and numbers and percentages as appropriate.

The EHF criteria for triptan non-response cannot be completely reproduced from the present data, as this would require information from single attacks, the number of treated attacks, and separate information on pain intensity and non-pain symptoms. For the purpose of the present analysis, we defined a non-response to (failure of) a specific triptan as either (1) previous use of the triptan discontinued because of side effects or no effect/insufficient effect or (2) current use of the triptan (at the time of the analyzed visit) with efficacy or tolerability rated less than “good”. If the same triptan was mentioned more than once, the most recent entry was used. A responder to a current acute medication was defined as a patient who used the acute medication at the time of the analyzed visit and had rated both efficacy and tolerability as “very good” or “good”. Discontinuation for other reasons (of a previously used triptan) included all reasons other than side effects or no effect/insufficient effect (see above). Contraindications to triptans were not analyzed in the present study.

Statistical group comparisons between triptan responders and different levels of non-response were performed with Kruskal-Wallis ANOVA for continuous data and Fisher Exact test for nominal data, followed by Bonferroni-corrected posthoc tests (Mann Whitney U tests or pairwise Fisher Exact tests) as appropriate.

## Results

The analysis was based on 2284 adult migraine patients who had participated in 1 to 14 visits (2.7 ± 2.1) within the DMKG Headache Registry. Characteristics are listed in Table 1. Of these 2284 patients, 1606 (70.3%) were previous or current triptan users. A detailed patient disposition with respect to triptan failure, response, and discontinuation for other reasons is given in Supplementary Table 1.

**Table 1:** Characteristics of the study population (*n* = 2284)

**Demographics**	
Age	39.4 ± 12.8
Sex	
- Female	1950 (85.4%)
- Male	329 (14.4%)
- Diverse	5 (0.2%)
**Headache characteristics**	
Diagnosis	
- Migraine without aura	1082 (47.4%)
- Migraine with aura	442 (19.3%)
- Migraine with and without aura	173 (7.6%)
- Chronic migraine	587 (25.7%)
Headache days per month^a^	12.3 ± 8.2
Severe headache days per month^a^	6.3 ± 5.7
Acute medication days per month^a^	6.7 ± 5.4
Headache intensity [0–10]^a^	5.6 ± 2.0
Headache duration [years]	19.4 ± 14.0
MIDAS score [0–279]	39.6 ± 47.1
Current preventive migraine medication	1215 (53.2%)
**Current or previous triptan use**	
Current or previous use of any triptan	1606 (70.3%)
Sumatriptan oral	729 (31.9% / 45.4%^b^)
Sumatriptan nasal	39 (1.7% / 2.4%^b^)
Sumatriptan subcutaneous	70 (3.1% / 4.4%^b^)
Rizatriptan	594 (26.0% / 37.0%^b^)
Naratriptan	483 (21.1% / 30.1%^b^)
Zolmitriptan oral	255 (11.2% / 15.9%^b^)
Zolmitriptan nasal	164 (7.2% / 10.2%^b^)
Eletriptan	116 (5.1% / 7.2%^b^)
Almotriptan	65 (2.8% / 4.0%^b^)
Frovatriptan	37 (1.6% / 2.3%^b^)

Values are mean ± SD or numbers and percentages with respect to the total population (*n* = 2284)

^a^Average of past 3 months. MIDAS, migraine disability assessment score

^b^For single triptans, percentages are also given with respect to the population of current or previous triptan users (*n* = 1606)

### Triptan responders and non-responders

**Fig. 1:**
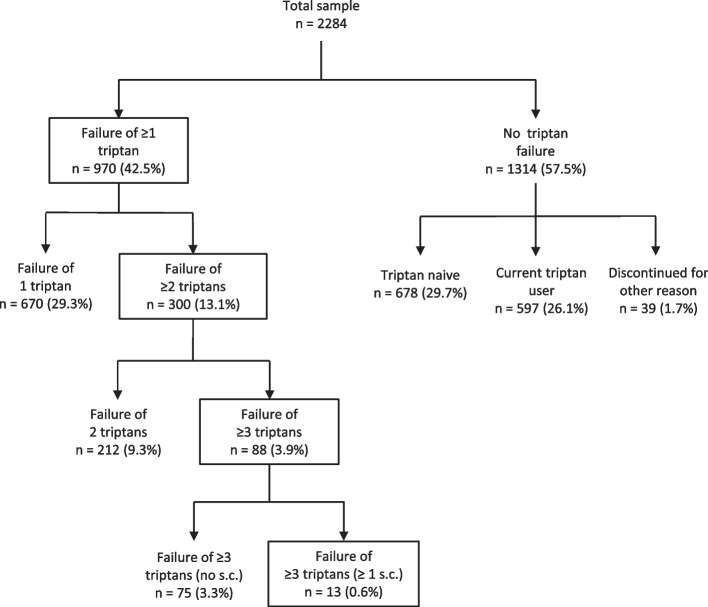
Triptan failure and no failure subgroups

Figure 1 illustrates the numbers of patients with triptan failure. 970 patients (42.5% of the total population) had failed ≥ 1 triptan, 300 (13.1%) had failed ≥ 2 triptans (EHF ‘triptan resistant’), 88 patients (3.9%) had failed ≥ 3 triptans and 13 patients (0.6%) had failed ≥ 3 triptans, including a subcutaneous formulation (EHF ‘triptan refractory’). It must be noted that only 70 patients had tried a subcutaneous triptan (Table 1). A detailed disposition according to triptan failures is given in Supplementary Fig. 1. Please note that triptan non-response (failure) as defined here means efficacy failure, tolerability failure, or both.

Regarding triptan response, 854 patients responded to at least one triptan (37.4% of the total population and 53.2% of the 1606 who ever used a triptan). More specifically, response to at least one triptan was found in 506 (51.7%) of the 978 patients who tried exactly one triptan, in 241 (56.4%) of the 427 patients who tried exactly 2 triptans and in 107 (53.2%) of the 201 patients who tried ≥ 3 triptans.

Clearly, even in the case of failure of 1, 2 or more triptans, there is still a chance that the patient might respond to another triptan, if tried. We estimated probabilities of response to (additional) triptans. As stated above, 51.7% (506 of 978 patients) who tried exactly 1 triptan responded to this triptan. For patients who tried exactly 2 triptans (*n* = 427) and failed 1 of these, the probability to respond to the other one was 45.0% (149 of 331 patients). For patients who tried exactly 3 triptans (*n* = 130) and failed 2 of these, the probability to respond to the third was 38.9% (35 of 90). For patients who tried ≥ 4 triptans and failed 3 of these, the probability to respond to an additional triptan was 29.2% (12 of 41). However, it has to be noted that the order of triptan trials was not known and that only part of the patients with 1, 2 or 3 triptan failures even tried an additional triptan.

Therefore, even EHF ‘triptan resistant’ patients may respond to additional triptans, if tried. In the present sample, of the 300 patients who failed ≥ 2 triptans, 147 tried at least one additional triptan, and 63 (42.8% of those who tried and 21.0% of all 300 patients who failed ≥ 2 triptans) responded to at least one additional triptan. Thus, the number of patients who failed ≥ 2 triptans and who also had no response to any additional triptan that was tried eventually was 237 (10.4% of the total population). If we also consider patients who did respond to other analgesics (non-opioid analgesics and combination analgesics without opioid), the number was further reduced to 176 patients (7.7% of the total population). These data are displayed in Table 2 also for the other categories of triptan failure.

**Table 2:** Triptan failure subgroups, including patients responding to an additional triptan or other acute medication

	**Failure of…**			
	… ≥ 1 triptan	… ≥ 2 triptans	… ≥ 3 triptans	… ≥ 3 triptans, including ≥ 1 s.c. triptan
…and no additional requirements	970 (42.5%)	300 (13.1%)^b^	88 (3.9%)	13 (0.6%)^c^
…and no response to any triptan attempted	713 (31.2%)	237 (10.4%)	74 (3.2%)	12 (0.5%)
…and no response to any acute medication attempted^a^	547 (23.9%)	176 (7.7%)	56 (2.5%)	10 (0.4%)

Absolute numbers and percentages with respect to the complete sample are given

^a^Including triptans, non-opioid analgesics and combination analgesics (without opioid component)

^b^Meeting the EHF criteria for ‘triptan resistant’

^c^Meeting the EHF criteria for ‘triptan refractory’

### Comparison of patients with and without triptan non-response

**Fig. 2:**
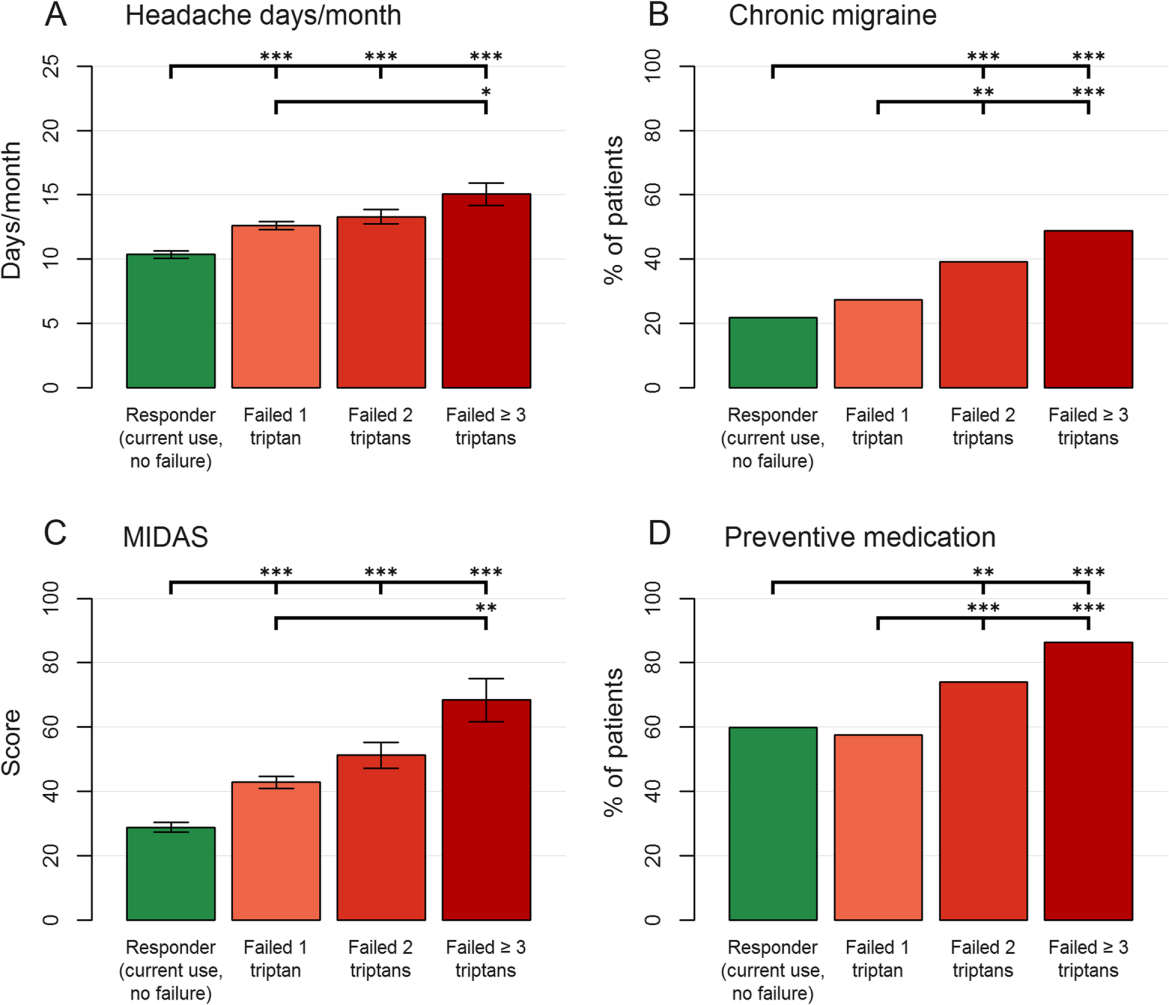
Comparison between triptan failure and no failure subgroups. MIDAS, Migraine disability assessment. */**/*** indicate significance at the *p* < 0.05/0.01/0.001 level in the Bonferroni-corrected post-hoc test

Compared to triptan users without a history of triptan failure, non-responders to one or several triptans were significantly more severely affected by their migraine (more chronic migraine diagnoses, higher headache and severe headache frequencies, higher migraine disability assessment (MIDAS [14]) scores) (Fig. 2, Supplementary Table 2). These parameters also increased with increasing triptan non-response (to 1, 2, or ≥ 3 triptans), although not all pairwise comparisons reached statistical significance. The proportion of patients using preventive migraine medication also increased significantly with increasing triptan non-response. Patients with triptan non-response were slightly younger than patients with current use and no failure. Importantly, there was no significant difference in the proportion of patients with acute medication use on ≥ 10 days/months between groups.

### Different triptan substances and formulations

**Fig. 3:**
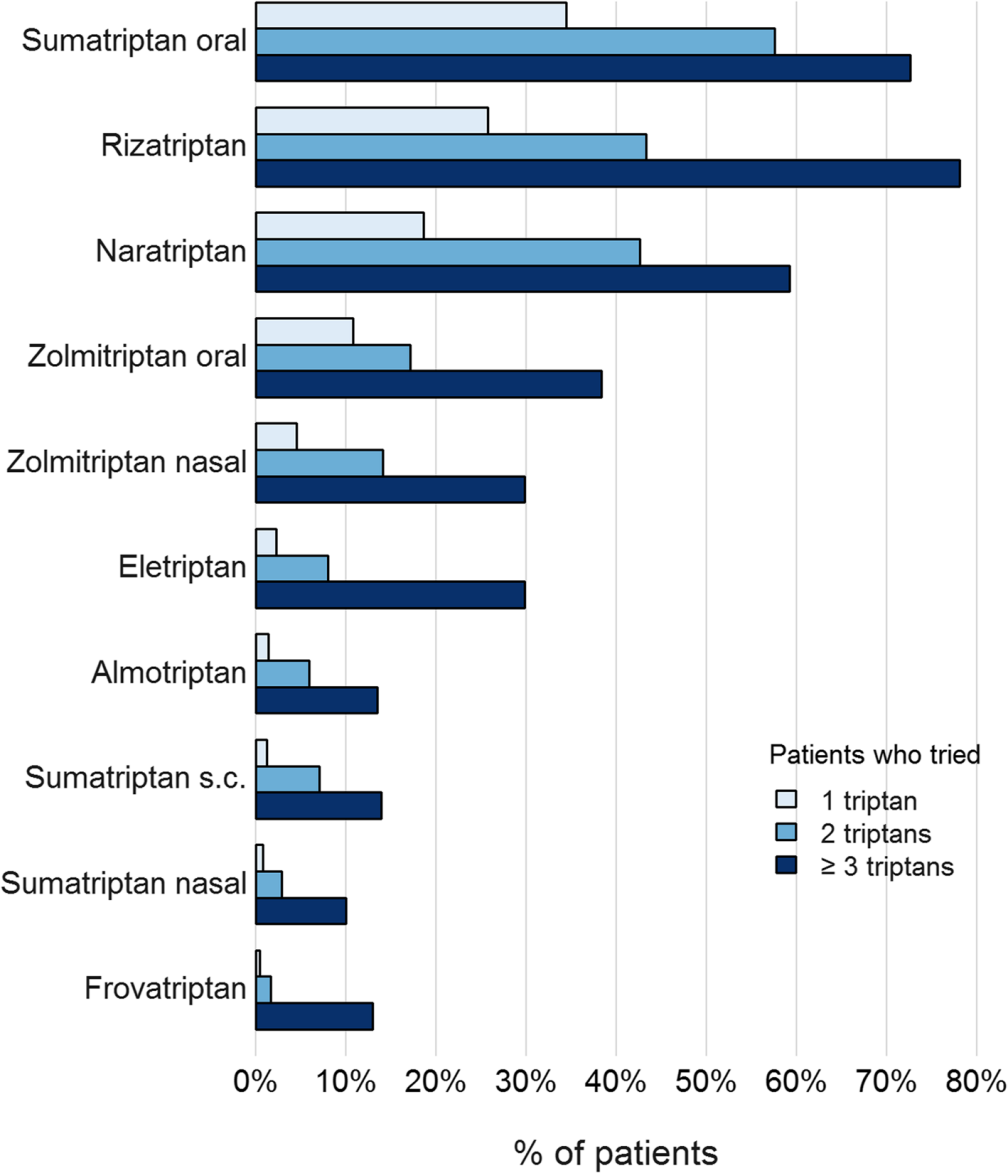
Proportions of patients having used or using specific triptans within the study population

Figure 3 illustrates which triptans were tried by patients who attempted therapy with 1, 2 or ≥ 3 triptans (see Supplementary Table 3 for exact numbers). The most frequently used triptans were sumatriptan (oral), rizatriptan, naratriptan und zolmitriptan (oral). The largest increases between the “tried 2 triptans” and the “tried ≥ 3 triptans” groups were found for rizatriptan (+34.8%), eletriptan (+21.9%), oral zolmitriptan (+21.2%) and naratriptan (+16.6%). Among those who had tried ≥ 3 triptans, parenteral triptans accounted for relatively low numbers (28.4% for nasal zolmitriptan, 14.2% for subcutaneous sumatriptan, 10.0% for nasal sumatriptan).

**Fig. 4:**
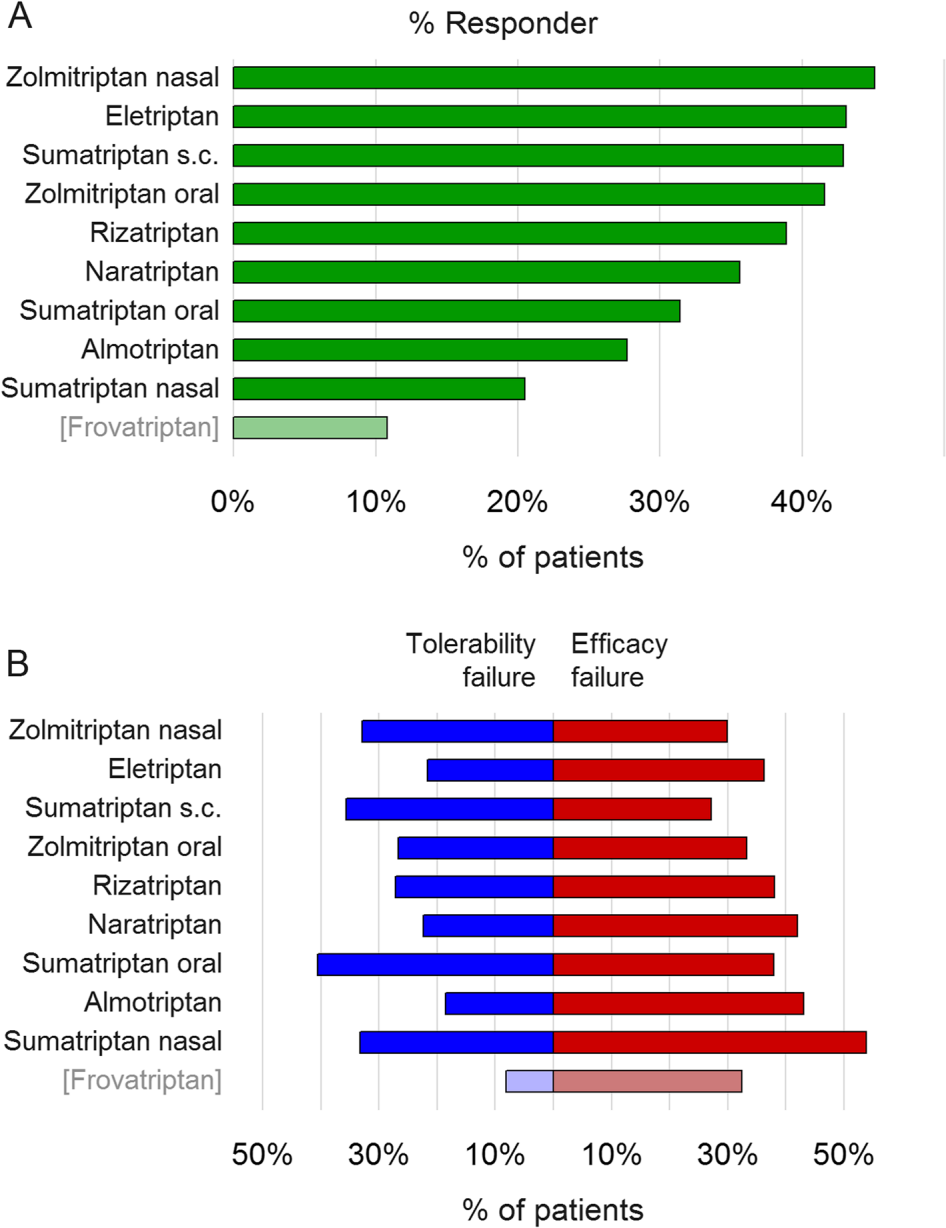
Frequency of response and reasons for failure of specific triptans. **A** Triptan responders. For each triptan, bars illustrate percentages of patients having responded to this triptan with respect to all patients who tried this triptan. **B** Reasons for triptan failure. Bars indicate percentages of patients having failed this triptan for tolerability or efficacy reasons with respect to all patients who tried this triptan, respectively. Note that a patient can have both tolerability and efficacy failure for the same triptan. Percentages for frovatriptan appear artificially low because > 50% of patients discontinued frovatriptan for other reasons. Complete data including discontinuation for other reason are given in Supplementary Table 4

Figure 4A illustrates response to specific triptans, showing that zolmitriptan (nasal and oral), eletriptan and sumatriptan (subcutaneous) had the largest proportions of patients with a response (meaning the triptan was both effective and tolerable). Figure 4B explores the reasons for triptan failure (efficacy vs. tolerability failure, see Supplementary Table 4 for exact numbers). Least efficacy failures were found for sumatriptan (subcutaneous), zolmitriptan (nasal and oral) and eletriptan, while least tolerability failures were found for almotriptan, eletriptan, naratriptan, zolmitriptan (oral) and rizatriptan. Sumatriptan (oral) had the largest number of tolerability failures and sumatriptan (nasal) had the largest number of efficacy failures. Responder and failure percentages for frovatriptan appear artificially low as a large number of patients discontinued this triptan for ‘other reasons’ (54.1%, Supplementary Table 4), likely due to national reimbursement and unavailability issues (see discussion). This number was also somewhat elevated for almotriptan (16.9%) with respect to the other triptans (generally < 10%).

## Discussion

Main result of the present study is that in the present sample of migraine patients, 42.5% had failed ≥ 1 triptan, 13.1% had failed ≥ 2 triptans and 3.9% had failed ≥ 3 triptans. Subtracting patients who found another effective and tolerable acute medication (e.g. another triptan or non-opioid analgesic) reduced these numbers to 21.5%, 7.1% and 2.3%, respectively. It further resulted that migraine patients who failed one or several triptans were significantly more severely affected by their migraine than triptan users without non-response. On a single substance/formulation level, largest overall responder rates were found for nasal and oral zolmitriptan, eletriptan and subcutaneous sumatriptan. It is important to note that the definition of response used here requires the triptan to be both effective and tolerable.

### Frequency of triptan failure

Present data show that 42.5% of the patients had failed ≥ 1 triptan and 13.1% had failed ≥ 2 triptans. It must be considered that these percentages are with respect to the total population, that includes 29.7% of patients never having tried a triptan. These results are slightly higher compared to a pooled analysis of rimegepant studies that showed insufficient response to ≥ 1 triptan and to ≥ 2 triptans in 35.2% and 9.3% of the study population, respectively [8]. It has been emphasized that failure of one triptan does not mean failure of every triptan [1], and the EHF definition accounts for this fact by demanding non-response to at least two triptans for ‘triptan resistance’. In view of the availability of new and effective oral drugs, requiring failure of 2 triptans before switching to a new drug class such as ditans or gepants (as proposed by the EHF [10]) seems reasonable.

However, in the present study, a significant number of patients with failure of two triptans responded to another triptan. Patients having tried 3 triptans and failed 2 of them had a 38.9% probability to respond to the third. However, it has to be noted that only 49% of the patients with failure of 2 triptans even tried a third triptan, and there might be a reason for this (e.g. partial response might prompt additional trials) so that the real response rates to a third triptan after failure of 2 might be lower. Nonetheless, the overall percentage of patients having failed 2 triptans (13.1%) was reduced to 10.4% when subtracting those who found an effective triptan during additional trials. Therefore, trying another triptan, especially one with a high response rate, can be an alternative to switching drug class for part of the patients. In the present study, highest response rates were found for zolmitriptan (oral and nasal), eletriptan (oral) and sumatriptan (subcutaneous) (see below). Obviously, it is also important to optimize treatment with a specific triptan (use early in the attack, appropriate dosing, treatment of several attacks) before declaring failure of this triptan [1, 2]. Finally, part of the patients with failure of 2 triptans respond to non-opioid or combination analgesics, so making sure that an adequate trial with these substances has been made is important.

Although it has the largest efficacy according to clinical studies [2], and is also among the triptans with the highest responder rates within the present study, requiring a trial with subcutaneous sumatriptan before switching to a novel oral drug class does not seem reasonable. Many patients prefer oral medication, and subcutaneous sumatriptan can be more expensive than the novel drugs. E.g., in Germany, at the time of this publication, a sumatriptan s.c. 6 mg dose is 12.5× the cost of an oral triptan dose, and 1.6× the cost of a lasmiditan 100 mg dose. Gepants are not yet available in Germany. Indeed, present data show that subcutaneous sumatriptan was prescribed only in a minority of patients (3.1% total and 13.9% of those having tried ≥ 3 triptans).

Clearly, the present results have to be interpreted in the context of specialized headache care, as DMKG Headache Registry data stem from headache centers and practices with a special interest in headache. This is also reflected by the large proportion of chronic migraine diagnoses (25.7%), the high average number of headache days per months (12.3 ± 8.2) and MIDAS score (39.6 ± 47.1; a score above 20 indicates severe migraine-related disability [14]). As failure of acute medication is one reason for referring patients to secondary/tertiary headache care, proportions of triptan resistant patients likely are smaller in primary care.

Present data (Supplementary Fig. 1) also show that a small proportion of patients switch to a second or even third triptan without failure of the first triptan. This might be due to economic considerations, availability, physician or patient preferences, the hope to achieve even better efficacy and tolerability, or use of more than one effective and tolerable triptan (e.g. an oral triptan for most attacks and a parenteral formulation for escalation therapy).

### Patients with triptan failure more severely affected by their migraine

Previous studies have reported that triptan non-responders have more severe migraine (higher frequency and intensity, more accompanying symptoms, higher disability) than triptan responders [1, 15], and a previous DMKG Registry analysis showed that migraine patients with higher headache frequency have lower acute medication efficacy [16]. The present data expand these results, showing that migraine severity (headache and severe headache frequency, a chronic migraine diagnosis, headache intensity) and associated disability (MIDAS) further increased with increasing level of triptan failure (Fig. 2). While headache frequency increased by a factor of 1.4 from the ‘current use, no failure’ group to the ‘failure of ≥ 3 triptans’ group, MIDAS scores increased by 2.4. Thus, triptan failure disproportionately increased migraine-related disability, likely because insufficiently treated migraine attacks cause more disability than sufficiently treated attacks. In addition, insufficient acute migraine treatment increases the risk for migraine chronification [17]. Together, these results show that patients with failure of one or several triptans need our special attention. Acute treatment optimization strategies may include: treating early during the attack, using a different dose or formulation, switching to a different triptan, combining triptans with non-steroidal anti-inflammatory drugs (NSAIDs, e.g. naproxen) or switching to a different class of acute medication [1, 2]. These patients also need close follow-up to further adjust treatment if necessary. In addition, starting a migraine preventive medication may improve efficacy of the acute medication [11, 18]. In the present study, the use of migraine preventive medication was high, and even higher in patients with non-response to ≥ 2 triptans (Fig. 2). This shows that this strategy was often used in the present setting of specialized migraine care, but that the migraine burden of these patients nonetheless remained high. In addition, biobehavioural migraine preventive treatments (e.g. relaxation techniques, physical activity, biofeedback) should be implemented in all patients needing a migraine preventive medication. In the present data, triptan non-responders also were slightly younger than patients without triptan failure. It seems unlikely that this is a sign of better triptan response with increasing age. A possible explanation might be that patients with triptan non-response are referred to specialized care earlier than patients with a good response.

### Comparison between different triptans and formulations

Consistent with German health insurance data [3], oral formulations of sumatriptan, rizatriptan, naratriptan and zolmitriptan were most frequently used as the first triptan (Fig. 3). In patients trying a third triptan, the pattern shifted towards rizatriptan, eletriptan, zolmitriptan (oral) and naratriptan. Use of rizatriptan or eletriptan after first triptan failure has been reported before [8]. As rizatriptan and eletriptan according to clinical studies are the most effective oral triptans and naratriptan is among the most tolerable triptans, this indicates reasonable choice of treatments [19, 20].

Regarding responder rates and reasons for non-response (Fig. 4), our results are generally consistent with the results of clinical trials [2, 19, 20]. Several points merit further discussion. Oral sumatriptan, the most frequently used triptan, showed a relatively low proportion of responders and a high proportion of tolerability failures. In clinical studies, sumatriptan ranged among the triptans with medium efficacy, which may partly explain these results [19]. Alternatively, failure of sumatriptan, the most frequently used triptan in Germany, may prompt referral to specialized headache care, resulting in patients with a poor response to sumatriptan being overrepresented in the present study population. Second, nasal sumatriptan had the largest number of efficacy failures. This result might not have been expected from early clinical trial data [21] but later studies indeed showed limited efficacy [22]. It might also be worth mentioning that some oral triptans (eletriptan and oral zolmitriptan) achieved responder rates very similar to the strong and fast acting parenteral triptans (nasal zolmitriptan and subcutaneous sumatriptan). It has been emphasized before that patient efficacy ratings comprise more than 2 h pain-free rates [23]. In addition, response in the present study encompassed both efficacy and tolerability. Finally, > 50% of patients discontinued frovatriptan for “other” reasons, likely because frovatriptan is expensive and (different from other triptans) only partially reimbursed by German health care. In addition, there have been availability issues. This makes frovatriptan data difficult to interpret.

### Limitations

Determination of triptan failure under real world conditions might easily be biased in both directions. A specialized headache care population (as represented by the DMKG Headache Registry) likely has higher rates of triptan resistance, both because triptan resistance might be more frequent in this population and because patients have higher odds to be offered several triptans in the first place. There could also be a bias towards patients having failed the most frequently used triptans (sumatriptan, rizatriptan, naratriptan) that might have affected the response rates of these substances. In contrast, true rates of failure of ≥ 2 triptans likely would be underestimated in a primary care setting where some patients may not be offered a second triptan after failure of the first. Even in the population analyzed here, 19.7% of the patients had failed their first triptan and had not (yet) tried a second one. The number of visits within the DMKG Headache Registry varied between 1 and 14 (2.7 ± 2.1), therefore not all patients had already undergone acute treatment optimization in specialized care. Thus, the present results have to be taken as a snapshot giving a coarse estimation of how frequent triptan resistance is in a specialized headache care setting. Second, it must be considered that triptan failure in the present study was based on retrospective patient self-report. Especially, past medications used before the first visit within the registry were assessed retrospectively at the first visit, possibly leading to recall bias and to incomplete reporting. Also, there is no way to know if patients observed the rules of early dosing and treating several attacks before declaring failure of a specific triptan. Regarding dosages, our previous results show that triptan underdosing is very rare in the present sample [16]. Nonetheless, choice of a low vs. high dose within the range of recommended dosages might affect triptan efficacy and tolerability. This was not analyzed here because numbers in some of the dosage subgroups were too small.

## Conclusions

Within the limits of a real-world setting, the present data show that a substantial proportion of patients in our specialized headache care setting failed one or several triptans. Migraine severity, especially migraine-related disability, increased significantly with the number of triptan failures. Therefore, the most important message from this study is that these patients need our special attention. Acute treatment optimization may include observing acute medication rules (early treatment with a sufficient dose), switching to a triptan with a high response probability, or switching to another acute medication class such as ditans or gepants.

### Supplementary Information

**Additional file 1: Supplementary Table 1.** Patient disposition with respect to triptan trials, failures, responses and discontinuations for other reasons. **Supplementary Table 2.** Characteristics of triptan responder and failure categories. **Supplementary Table 3.** Use of specific triptans in the study population. **Supplementary Table 4.** Specific triptans: Proportions of patients with response and failure, and reasons for failure.

**Additional file 2: Supplementary Figure 1. **Patient disposition with respect to triptan failures.

## Data Availability

The datasets analysed during the current study are available from the German Migraine and Headache Society on reasonable request. Please note that access to data principally suitable for conducting additional analyses must be reviewed by the Headache Registry’s Scientific Steering Committee.

## Appendix

The following centers have contributed data to the present analysis: Dr. Gendolla, Essen; Dr. Marziniak, München; Dr. Goßrau, Dresden; Dr. Rambold, Mühldorf, Dr. Kukowski, Hildesheim, Dr. Peikert, Bremen, Dr. Weber, Fürstenfeldbruck; Dr. Friedrich, Ravensburg; Dr. Förderreuther, München; Dr. Thilmann, Mannheim; Dr. Malzacher, Reutlingen; Dr. Menekes, Stuttgart, Dr. Ruscheweyh, München, Dr. Lewis, Stuttgart, Dr. Weber, Erlangen, Dr. Ermeling-Heuser, Bonn, Dr. Fleischmann, Greifswald, Dr. Rimmele, Rostock, Dr. Erbacher, Straubing, plus 3 centers that in the meantime have migrated or stopped recruitment.

## Abbreviations

AHS: American Headache Society
CGRP: Calcitonin gene-related peptide
DMKG: Deutsche Migräne- und Kopfschmerzgesellschaft (German headache and migraine society)
EHF: European Headache Federation
EMA: European Medicines Agency
FDA: Food and Drug Administration
ICHD-3: International classification of headache disorders, 3^rd^ edition
MIDAS: Migraine disability assessment score
