# Supplementary material for: Triptan non-response in specialized headache care: cross-sectional data from the DMKG Headache Registry
Source: J Headache Pain. 2023 Oct 10;24(1):135. doi: 10.1186/s10194-023-01676-0 (PMC10563311; doi:10.1186/s10194-023-01676-0)
Supplement: Supplementary file 1 — Additional file 1: Supplementary Table 1. Patient disposition with respect to triptan trials, failures, responses and discontinuations for other reasons. Supplementary Table 2. Characteristics of triptan responder and failure categories. Supplementary Table 3. Use of specific triptans in the study population. Supplementary Table 4. Specific triptans: Proportions of patients with response and failure, and reasons for failure. [file 10194_2023_1676_MOESM1_ESM.pdf]

Supplementary Table 1. Patient disposition with respect to triptan trials, failures, responses and discontinuations for other reasons.

		**Triptans failed**			
**Triptans tried**	n	0	1	2	≥3
0	678	678	-	-	-
1	978	529	449	-	-
2	427	96	178	153	-
≥3	201	11	43	59	88
		**Triptans with response**			
**Triptans tried**	n	0	1	2	≥3
0	678	678	-	-	-
1	978	472	506	-	-
2	427	186	196	45	-
≥3	201	94	71	27	9
		**Triptans discontinued for other reason**			
**Triptans tried**	n	0	1	2	≥3
0	678	678	-	-	-
1	978	849	35	-	-
2	427	136	83	4	-
≥3	201	24	43	15	6

Supplementary Table 2. Characteristics of triptan responder and failure categories

	No triptan failure	Failure of…			Statistics
	Current triptan use	… 1 triptan	… 2 triptans	… ≥3 triptans	
n	597	670	212	88	
Age	41.8 ± 12.4	39.8 ± 12.5*	39.1 ± 13.6*	38.3 ± 12.2	**H = 14.4**<br>**P = 0.002**
Sex					P = 0.161
- Female	510 (85.4%)	590 (88.1%)	196 (92.5%)	78 (88.6%)	-
- Male	84 (14.1%)	79 (11.8%)	16 (7.5%)	10 (11.4%)	-
- Diverse	3 (0.5%)	1 (0.1%)	0	0	-
Diagnosis					**P < 0.001**
- Migraine without aura	332 (55.6%)	312 (46.6%)*	68 (31.6%)$^{***,\$}$	30 (34.1%)	**P < 0.001**
- Migraine with aura	90 (15.1%)	120 (17.9%)	39 (18.4%)	10 (11.4%)	P = 0.408
- Migraine with and without aura	45 (7.5%)	55 (8.2%)	23 (10.8%)	5 (5.7%)	P = 0.489
- Chronic migraine	130 (21.8%)	183 (27.3%)	83 (39.2%)$^{***,\$\$}$	43 (48.9%)$^{***,\$\$\$}$	**P < 0.001**
Other headache & treatment characteristics					
Headache days per month[1]	10.4 ± 7.0	12.6 ± 8.2***	13.3 ± 8.3***	15.0 ± 8.3$^{***,\$}$	**H = 48.8**<br>**P < 0.001**
Severe headache days per month[1]	5.2 ± 4.5	6.6 ± 5.7***	7.7 ± 6.5***	7.7 ± 5.8***	**H = 42.5**<br>**P < 0.001**
Acute medication days per month[1]	6.6 ± 4.5	7.2 ± 5.4	7.2 ± 5.1	7.5 ± 4.5	H = 6.3<br>P = 0.100
Acute medication on ≥10 days/month	130 (21.7%)	178 (26.5%)	49 (23.1%)	24 (27.3%)	P = 0.424
Headache intensity [0-10][1]	5.5 ± 1.9	5.6 ± 2.0	6.0 ± 1.8*	5.9 ± 2.2	**H = 10.4**<br>**P = 0.016**
Headache duration [years]	21.9 ± 14.0	21.1 ± 13.8	22.0 ± 13.9	20.7 ± 12.8	H = 1.5<br>P = 0.676
MIDAS score [0-279]	28.9 ± 36.0	44.2 ± 49.3***	52.7 ± 59.1$^{***,\$\$}$	68.7 ± 62.3***	**H = 93.2**<br>**P < 0.001**
Preventive migraine medication	357 (59.8%)	386 (57.6%)	157 (74.1%)$^{**,\$\$\$}$	74 (86.4%)$^{***,\$\$\$}$	**P = 0.03**

Statistical comparisons between groups were performed, and significant results were marked in bold. Kruskal Wallis ANOVA and Fisher's Exact test were used as omnibus tests. Results of Bonferroni-corrected post-hoc tests are given as follows: $^{*/**/***}$, p<0.05/0.01/0.001 for comparison with the first group (no failure, current triptan use); $^{\$/\$\$/\$\$\$}$, p<0.05/0.01/0.001 for comparison with the second group (failure of 1 triptan)

Supplementary Table 3. Use of specific triptans in the study population

Drug & formulation		Total	Patients who tried …		
			… 1 triptan	… 2 triptans	… ≥3 triptans
Number of Patients		2284	978	427	201
Sumatriptan	oral	729 (31.9%)	337 (34.5%)	246 (57.6%)	146 (72.6%)
	nasal	39 (1.7%)	7 (0.7%)	12 (2.8%)	20 (10.0%)
	s.c.	70 (3.1%)	12 (1.2%)	30 (7.0%)	28 (13.9%)
Rizatriptan	oral	594 (26.0%)	252 (25.8%)	185 (43.3%)	157 (78.1%)
Naratriptan	oral	483 (21.1%)	182 (18.6%)	182 (42.6%)	119 (59.2%)
Zolmitriptan	oral	255 (11.2%)	105 (10.5%)	73 (17.1%)	77 (38.3%)
	nasal	164 (7.2%)	44 (4.5%)	60 (14.1%)	60 (29.9%)
Eletriptan	oral	116 (5.1%)	22 (2.2%)	34 (8.0%)	60 (29.9%)
Almotriptan	oral	65 (2.8%)	13 (1.3%)	15 (5.9%)	27 (13.4%)
Frovatriptan	oral	37 (1.6%)	4 (0.4%)	7 (1.6%)	26 (12.0%)

Percentages refer to the number of patients in the respective column.

Supplementary Table 4. Specific triptans: Proportions of patients with response and failure, and reasons for failure

						Reasons for failure	
		**Total**	**Response**	**Failure**	**Discontinued for other reason**	**Efficacy failure**	**Tolerability failure**
Sumatriptan	oral	729	229 (31.4%)	450 (61.7%)	50 (6.9%)	276 (37.9%)	295 (40.5%)
	nasal	39	8 (20.5%)	30 (76.9%)	1 (2.6%)	21 (53.8%)	13 (33.3%)
	s.c.	70	30 (42.9%)	37 (52.9%)	3 (4.3%)	19 (27.1%)	25 (35.7%)
Rizatriptan	oral	594	231 (38.9%)	321 (54.0%)	42 (7.1%)	226 (38.0%)	161 (27.1%)
Naratriptan	oral	483	172 (35.6%)	265 (54.9%)	46 (9.5%)	203 (42.0%)	108 (22.4%)
Zolmitriptan	oral	255	106 (41.6%)	123 (48.2%)	26 (10.2%)	85 (33.3%)	68 (26.7%)
	nasal	164	74 (45.1%)	78 (47.6%)	12 (7.3%)	49 (29.9%)	54 (32.9%)
Eletriptan	oral	116	50 (43.1%)	56 (48.3%)	10 (8.6%)	42 (36.2%)	25 (21.6%)
Almotriptan	oral	65	18 (27.7%)	36 (55.4%)	11 (16.9%)	28 (43.1%)	12 (18.5%)
Frovatriptan	oral	37	4 (10.8)	13 35.1%)	20 (54.1%)	12 (32.4%)	3 (8.1%)

Please note that a patient can have both efficacy and tolerability failure to the same triptan.

Discontinuation of triptans for other reason includes: found better medication, discontinuation advised by physician, not needed anymore because of headache improvement, other.

The exceptionally high (>50%) proportion of patients who discontinued frovatriptan for “other” reasons is most likely explained by the fact that frovatriptan is expensive and (different from other triptans) only partially reimbursed by German health care.
