# Supplementary figures and images for: Triptan non-response in specialized headache care: cross-sectional data from the DMKG Headache Registry
Source: J Headache Pain. 2023 Oct 10;24(1):135. doi: 10.1186/s10194-023-01676-0 (PMC10563311; doi:10.1186/s10194-023-01676-0)

Supplementary Figure 1

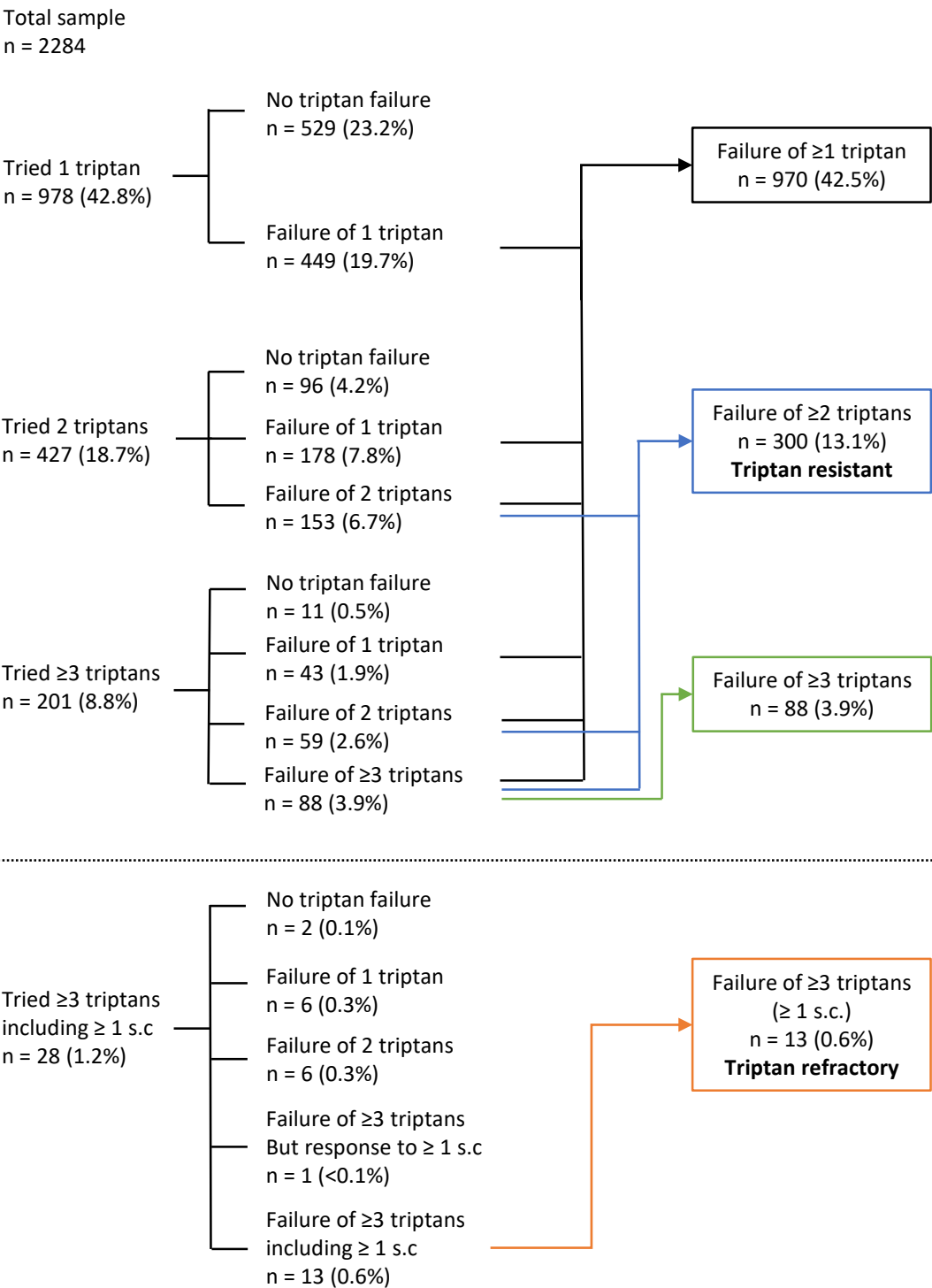

Supplement: Supplementary file 2 — Additional file 2: Supplementary Figure 1. Patient disposition with respect to triptan failures. [file 10194_2023_1676_MOESM2_ESM.pdf]
